# Variation and Distribution of L-A Helper Totiviruses in *Saccharomyces sensu stricto* Yeasts Producing Different Killer Toxins

**DOI:** 10.3390/toxins9100313

**Published:** 2017-10-11

**Authors:** Nieves Rodríguez-Cousiño, Pilar Gómez, Rosa Esteban

**Affiliations:** 1 Instituto de Biología Funcional y Genómica, IBFG-CSIC, Universidad de Salamanca, 37007 Salamanca, Spain; nievesrc@usal.es (N.R.-C.); pilargomez@usal.es (P.G.); 2 Escuela Politécnica Superior de Zamora, Universidad de Salamanca, 49029 Zamora, Spain

**Keywords:** yeast killer toxins, L-A totivirus, double-stranded RNA virus, *Saccharomyces sensu stricto*

## Abstract

Yeasts within the *Saccharomyces sensu stricto* cluster can produce different killer toxins. Each toxin is encoded by a medium size (1.5–2.4 Kb) M dsRNA virus, maintained by a larger helper virus generally called L-A (4.6 Kb). Different types of L-A are found associated to specific Ms: L-A in K1 strains and L-A-2 in K2 strains. Here, we extend the analysis of L-A helper viruses to yeasts other than *S. cerevisiae*, namely *S. paradoxus*, *S. uvarum* and *S. kudriavzevii*. Our sequencing data from nine new L-A variants confirm the specific association of each toxin-producing M and its helper virus, suggesting co-evolution. Their nucleotide sequences vary from 10% to 30% and the variation seems to depend on the geographical location of the hosts, suggesting cross-species transmission between species in the same habitat. Finally, we transferred by genetic methods different killer viruses from *S. paradoxus* into *S. cerevisiae* or viruses from *S. cerevisiae* into *S. uvarum* or *S. kudriavzevii*. In the foster hosts, we observed no impairment for their stable transmission and maintenance, indicating that the requirements for virus amplification in these species are essentially the same. We also characterized new killer toxins from *S. paradoxus* and constructed “superkiller” strains expressing them.

## 1. Introduction

Many yeasts produce killer toxins that kill other yeast of the same or different species. Most killer toxins are encoded on double-stranded RNA (dsRNA) viruses called M, which depend for their maintenance on a helper virus (generically called L-A) that provides the viral capsids where L-A and M are separately encapsidated. *Saccharomyces cerevisiae* virus L-A (ScV-L-A) is the type species of the *Totivirus* genus in the *Totiviridae* family and is widely distributed in yeasts (killer or non-killer) and other fungi [1]. The replication cycle of L-A (and its satellite M1) in the yeast cytoplasm of K1 killer strains has been thoroughly investigated [2]. The L-A (+) strand (4.6 Kb) contains two overlapping open reading frames (ORFs). The structural protein of the capsid (Gag) is encoded on the first ORF, while the viral RNA polymerase (Pol) is translated as a fusion protein (Gag-Pol) of the two overlapping frames by a −1 ribosomal frame shifting [3,4]. The so-called “killer system” has been extensively studied in *S. cerevisiae* [5,6] where four different killer toxins have been described: K1, K2, K28 and Klus, encoded on M1, M2, M28 and Mlus dsRNAs, respectively. Their sizes vary from 1.5 to 2.4 Kb [7,8,9,10]. Killer toxins are secreted glycoproteins with no amino acid sequence conservation among them that are translated initially as precursors called “preprotoxins” (pptox). Each type of killer strain is able to kill other killer-producing strains or non-killer yeasts, but it is immune to its own toxin. With respect to the killing mechanism, K1 causes pore formation in the cytoplasmic membrane of the sensitive cells and K2 also acts on yeast cell envelope; K28 blocks DNA synthesis and the mode of action of Klus is not known [11,12,13,14,15]. The genome organization of the M dsRNA satellites is similar: the positive strand contains an ORF in the 5′-end region that codes for the pptox, followed by an internal AU-rich region (also designated as internal PolyA), and a 3′-end non-coding region where the *cis* signals for encapsidation and replication by the helper virus RNA polymerase are present [10,16]. A few chromosomal ORFs with homology to Klus, K1 or K2 preprotoxins have been found in *S. cerevisiae* or in related yeasts, suggesting that the M viruses might have originated from host messenger RNAs that were encapsidated and replicated by the L-A helper virus RNA polymerase after acquiring sequences needed for both events, probably from the genome of the L-A virus itself [17]. Several L-A variants with an average of 74% nucleotide identity exist. They are associated with different M viruses: L-A, the first described, is the one present in most laboratory strains, either K1 or sensitive strains and appears to be the M1-specific helper virus in wild K1 strains. Other variants associated to specific Ms were named after their M satellite designations, thus L-A-lus is associated with Mlus, L-A-2 to M2 and L-A-28 to M28 [17,18,19]. The association of these distinct L-As with different M viruses suggests co-evolution and we have proposed a possible role of the toxin-producing M viruses selecting the L-A variants better fit to support them [18]. Killer systems based on dsRNA helper and satellite viruses have also been described in other yeasts such as *Ustilago maydis*, *Zygosaccharomyces bailii*, *Hanseniaspora uvarum* and *Torulaspora delbrueckii* [20,21,22], and in the *sensu stricto* group in *S. uvarum* and *S. paradoxus* [23,24,25].

The *Saccharomyces sensu stricto* taxon consists of seven natural species: *S. cerevisiae*, *S. uvarum*, *S. paradoxus*, *S. mikatae*, *S. kudriavzevii*, *S. arboricola* and *S. eubayanus* [26,27,28,29,30,31]. Additionally, there are hybrid species such as *S. pastorianus* (ex *S. carlbergensis*), a hybrid between *S. cerevisiae* and *S. eubayanus*; *S. bayanus*, a hybrid between *S. uvarum* and *S. eubayanus*; or hybrids between *S. cerevisiae* and *S. kudriavzevii*. The recent discovery of *S. eubayanus* [31] and the data from massive sequencing of genomes from *Saccharomyces* strains have clarified the taxonomy of the genus and the history of the species within this taxon [32]. Many species in the *sensu stricto* group are related with human activities: *S. cerevisiae* is mainly associated with bread baking, beer brewing or winemaking and *S. uvarum* is involved in cidermaking and winemaking at low temperatures. The hybrid species are also associated with fermentation processes. *S. paradoxus* is the closest relative of *S. cerevisiae* but, unlike baker’s yeast, it is present mainly in the wild, throughout the northern hemisphere associated with oak trees: bark, exudates or surrounding soil. It is distributed in geographical populations (European, American, Far Eastern and Hawaiian) clearly differentiated [33]. *S. paradoxus* has been isolated in substantial amounts from North America and Portugal together with *S. cerevisiae*, indicating that this species can also be present in natural environments [34,35]. In the later study *S. uvarum* and *S. kudriavzevii* were also isolated at low temperature. It is possible that yeasts from the oak trees habitat may have moved to flowers and ripened fruits and thereafter to fermented beverages [35]. Insects, such as social wasps, may have played an important role in *Saccharomyces* species dispersal and even in favoring hybridization between different species [36,37]. With respect to the killer toxins *S. cerevisiae* and *S. paradoxus* produce, data in the literature is somehow confusing: there are reports about the same toxin, i.e., K1 toxin, produced by strains belonging to either species [38], or cases in which the same *S. paradoxus* wild strain (e.g., strain T21.4) has been reported as producing different toxins [24,25].

Thus, in this work, our first aim was to clarify the type of killer toxins produced by *S. paradoxus*, as well as to determine which L-A helpers were supporting the M satellites encoding them. Our second aim was to study the possible horizontal cross-species transmission of killer viruses among *sensu stricto* members, mainly between *S. paradoxus* and *S. cerevisiae*. We successfully introduced different types of killer viruses in four *Saccharomyces* species by genetic crosses mimicking conditions in the wild, and analyzed the behavior of the transmitted viruses in the new host. Our data suggest that there is no impairment for maintenance in the new species, and that one of the main barriers for killer viruses transmission may be nuclear-mitochondrial incompatibility among species. Next, using the ease of genetic manipulation of *S. cerevisiae*, we have created a collection of “superkiller” strains secreting several killer toxins (originally produced by *S. paradoxus*) that will facilitate their further characterization. Finally, the analysis of the sequences of nine new L-A variants (from *S. paradoxus* or other *sensu stricto* yeasts) allowed us to get insights onto the evolution and relationship of the members of the L-A *totivirus* within the cluster.

## 2. Results

### 2.1. Killer Viruses from *S. paradoxus* Are Different from Those from *S. cerevisiae*

We have demonstrated the existence of different L-A variants in killer strains of *S. cerevisiae*, each one associated specifically to a toxin-encoding M dsRNA satellite. We tried to extend these findings to other toxin-producing yeasts within the *sensu stricto* cluster, namely *S. paradoxus*, since several strains of this species had been reported to carry K1, K28 or other viruses of yet unidentified nature. Data in the literature, however, was somehow controversial regarding some of the strains analyzed: e.g., strain Sp T21.4 (Table 1) was considered K1 by one group [24] or K28 by another [25].

**Figure 1:**
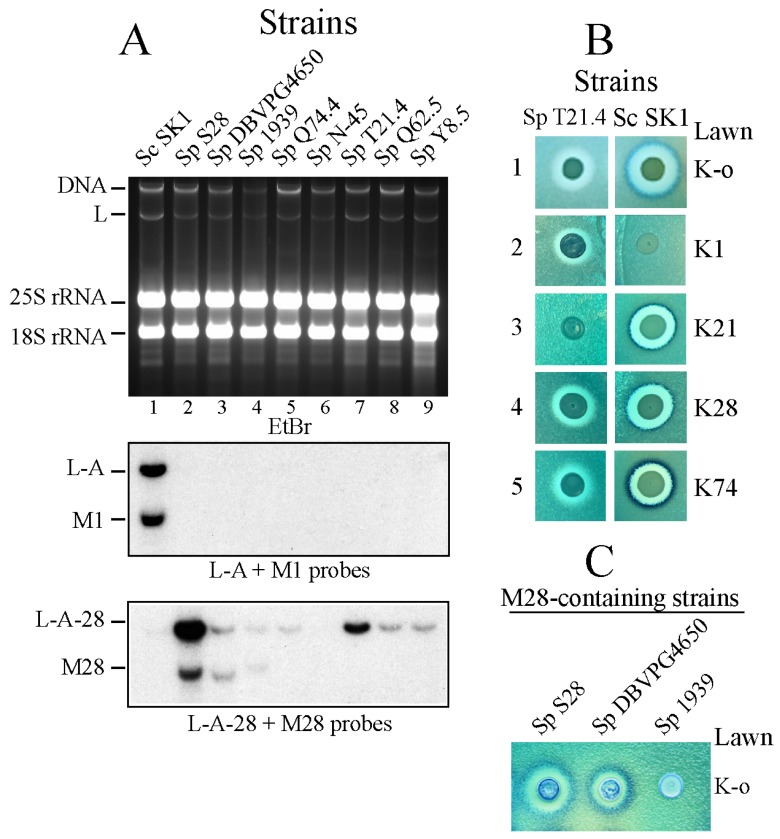
(**A**) Presence (or absence) of M1 or M28 virus in Killer strains of *S. paradoxus*. RNAs from a number of killer toxin-producing *S. paradoxus* (Sp) strains (lanes 2 to 9) and the *S. cerevisiae* (Sc) laboratory strain SK1 (lane 1) were separated on an agarose gel and transferred to a nylon membrane for Northern hybridization. The upper panel shows the ethidium bromide-stained gel (EtBr) with indications to the mobility of main nucleic acids. Below panels are autoradiograms of the Northern blot hybridized with a mixture of L-A- and M1-specific probes (middle) or L-A-28- and M28-specific probes (lower). (**B**) Killer activity of strains Sp T21.4 (left panels) and Sc SK1 (right panels) over lawns of a sensitive K-o strain (1) or four *S. cerevisiae* strains producing the indicated killer toxins: K1 (2), K21 (3), K28 (4) or K74 (5). Clear halos of growth inhibition in the lawn surrounding the central spots of cells indicate toxin activity. (**C**) Killer activity of the three *S. paradoxus* strains carrying M28 dsRNA variants analyzed in lanes 2, 3 and 4 of panel (**A**). The lawn is the same K-o strain as in (**B**).

In addition, strain Sp Q62.5 was assigned as a K1-producing strain or as a producer of an unknown different toxin [24,25]. In the cases cited, characterization was based on sensitivity (or resistance) to known tester strains belonging to the K1-, K2- or K28-type, or the positive amplification of RT-PCR products with oligonucleotides (oligos) designed according to the known four killer toxins of *S. cerevisiae*. Unfortunately, in certain cases both approaches seem to have led to wrong conclusions. Thus, we decided to use specific probes against the four M dsRNAs (M1, M2, Mlus or M28) and performed Northern hybridization with RNAs isolated from the same killer strains. According to our data, none of the *S. paradoxus* killer strains analyzed belong to the K2- or Klus-type, which seem to be confined to *S. cerevisiae* wine strains (not shown). The M1-specific probe recognized the RNA in the control *S. cerevisiae* SK1 strain but none of the *S. paradoxus* strains analyzed gave positive identification, indicating that they do not carry M1 viruses (Figure 1A).

Of the strains analyzed, there were four (Sp T21.4, Sp Q74.4, Sp Q62.5 and Sp Y8.5, Table 1) that had been considered K1 by one group [38] and one strain (Sp N-45) that had also been identified as K1 in other work [25]. To the best of our knowledge there are no *S. paradoxus* strains carrying M1 viruses. We did a search in the literature and only found K1 killers in *S. cerevisiae* strains from laboratory (or in yeast collections) that most likely derived by inbreeding from one (or few) original K1 strains analyzed by Makower and Bevan in the 1960s [39]. One of the original killer yeasts (NCYC 232, Table 1) was deposited in 1951 as Ex-American Yeast Foam by R. S. W. Thorne. According to data from a screening of killer character in yeast genera, most of the K1 strains detected were hybrids related to that strain [40]. We sequenced the genome of the L-A helper virus in strain NCYC 232 and also in the SK1 standard K1 killer and found them to have the same sequence deposited by Wickner’s [3] and Bruenn’s labs [41]. Thus, all the K1 killer strains positively identified carry the same helper virus (L-A) and belong to *S. cerevisiae* and not to *S. paradoxus* as claimed [24,25,38].

In the case of the M28-specific probe, the probe recognized the M28 control dsRNA from strain Sp S28 and in a lesser extent the M in strain Sp DBVPG4650, confirming previous data by Chang et al. [25] who reported that the latter strain carries an M28 variant with about 92% nucleotides (nt) identical to the one in Sp S28. Our probe, however, did not recognize the M dsRNA from strain Sp T21.4 (described by the same authors as carrying an M28 variant identical to that of Sp DBVPG4650). Thus, our data indicate that strain Sp T21.4 does not carry either M1 or M28-like virus (Figure 1A), but a different type of toxin-producing virus (which we named M21, see later). Indeed, K1 or K28 killer strains are sensitive to the toxin produced by Sp T21.4 in a bioassay (Figure 1B), indicating that they produce different toxins. The M28 probe also gave a weak signal to the M dsRNA of strain CECT1939 (CBS432), suggesting that it also carries an M28-type virus (see below). Since these three M28-containing strains apparently belong to two different species: *S. cerevisiae* (S28) and *S. paradoxus* (DBVPG4650 and CECT1939), we performed PCR amplification and sequencing of the 5.8S-internal transcribed spacers (ITS) rDNA regions of these three strains. Our data showed that strain S28 is in fact *S. paradoxus* and not *S. cerevisiae* as originally described [42]. Thus, the three M28 dsRNA carriers belong to the same species (*S. paradoxus*). Since strain CECT1939 appears to be non-killer (or very weak killer) in a standard bioassay (Figure 1C) we cloned and sequenced the M28 ORF of this strain (GenBank Acc. N° MF358735). We found nt changes in its sequence similar to those existing between S28 and DBVPG4650 M dsRNAs (about 95% identity to M28 in S28 and 92% to the M in strain DBVPG4650). The differences in its amino acid (aa) composition vary between 5% and 6% with respect to the other pptox. Some of the aa changes could affect its folding, as suggested by the disulfide bonding formation predicted by DIANNA [43]. In addition, the predicted N-glycosilation pattern is different as compared to the other two pptox, which are active (not shown). The three M28-carrying strains (as seen below) carry helper viruses with nt sequences quite similar, indicating a close evolutionary relationship.

With respect to the other killer types in the *S. paradoxus* strains analyzed in Figure 1A, strains Sp Y8.5 and Sp Q74.4 carry the same type of M dsRNA, which is different from the ones in Sp T21.4, Sp N-45 or Sp Q62.5 (Figure S1). We obtained the complete nucleotide sequences of these four new M dsRNAs [44] as mentioned in Methods, which code for pptox different from any of the previously known killer toxins. We named the M dsRNAs and the toxins they code for following designations of the *S. paradoxus* strains that produce them; thus the K21 toxin is encoded by M21, the RNA present in strain Sp T21.4, K45 is encoded by M45, present in strain Sp N-45, K74 encoded in M74 comes from strain Sp Q74.4, and K62 encoded in M62 comes from strain Sp Q62.5. In summary, at least five different killer toxins are produced by *S. paradoxus* strains and three by *S. cerevisiae*, and none of them seem to be present in strains from both species. Data from cross-killing assays with killer strains from these two species are presented in Table S1.

### 2.2. Different L-A Variants in *S. paradoxus* Strains

**Figure 2:**
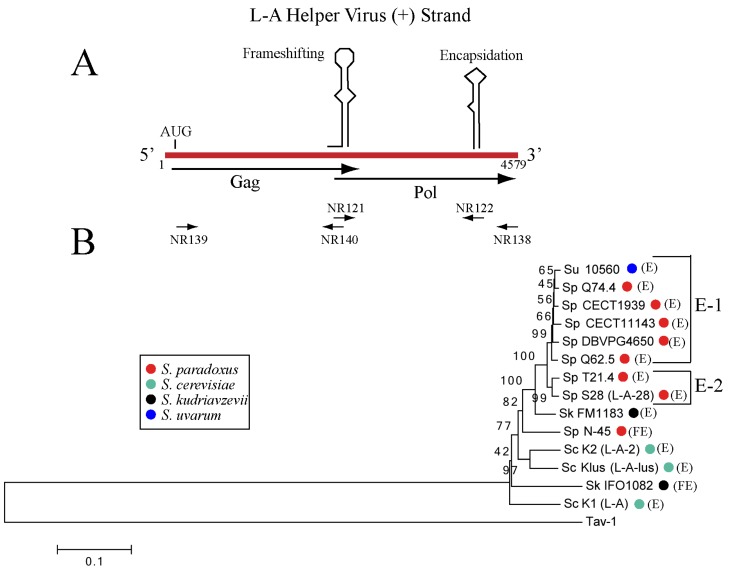
(**A**) Schematic diagram of L-A helper virus (+) strand genomic organization with two *cis* signals involved in a -1 translational frame shift and encapsidation (conserved in all the L-A helper viruses of *S. sensu stricto* species). The two ORFs for Gag and Pol are shown below. Oligos used for RT-PCR amplification and sequencing (see Methods) are indicated. (**B**) Dendrogram of the Gag-Pol amino acid sequences of 14 L-A variants analyzed in this work. The names and GenBank accession numbers are described in Methods. Each *Saccharomyces* species is indicated by distinct colored dots: *S. paradoxus* (Sp) red, *S. cerevisiae* (Sc) green, *S. kudriavzevii* (Sk), black and *S. uvarum* (Su), blue. In brackets their geographical location are indicated: European (E), or Far East (FE). Two subgroups of *S. paradoxus* European strains are indicated as E-1 or E-2 on the right. We used as outgroup the tuber aestivum (Tav-1) virus (GenBank HQ158596.1). The evolutionary history was inferred using the Neighbor-Joining method [45]. The optimal tree with the sum of branch length = 1.92844968 is shown. The percentage of replicate trees in which the associated taxa clustered together in the bootstrap test (1000 replicates) are shown next to the branches [46]. The tree is drawn to scale, with branch lengths in the same units as those of the evolutionary distances used to infer the phylogenetic tree. The evolutionary distances were computed using the Poisson correction method [47] and are in the units of the number of amino acid substitutions per site. The analysis involved 15 amino acid sequences. All positions containing gaps and missing data were eliminated. There were a total of 1504 positions in the final dataset. Evolutionary analyses were conducted in MEGA6 [48].

Once we clarified the type of toxins produced by *S. paradoxus*, we asked which helper viruses were supporting them. Northern analysis in Figure 1A indicated that they were different from L-A, and that some of them might be related to L-A-28. The frame shifting region and encapsidation signal (Figure 2A) are essential features of the L-A virus and are conserved in all L-A variants so far analyzed. Using oligos based on those sequences, we amplified by RT-PCR a 2.2-Kb fragment from all *S. paradoxus* strains and sequenced it.

Strains used are listed in Table 1. Most of them belong to European populations [33] and one comes from Far East (Sp N-45). In addition to seven *S. paradoxus* strains we also sequenced L-A viruses from two other species within the *sensu stricto* (reported as carrying L-A dsRNA though non-killer); one was *S. uvarum* (strains CECT1884 and CECT10560) and the other was *S. kudriavzevii* (strain IFO1802)*.* As described in Methods, we determined the complete nt sequences of all L-A variants, except for the first 20 nt of the 5′ end of the (+) strand. As expected, no sequence identical to that of L-A from *S. cerevisiae* K1 killer strains was found in the ten strains analyzed, confirming that the absence of M1 was correlated with the absence of L-A. The new L-A variants in these three *sensu stricto* species showed different degrees of variation. We found that *S. paradoxus* strain Y8.5 carried the same helper (and M) (Figure S1) as strain Q74.4. The same was true for the two *S. uvarum* strains analyzed. Thus, in our comparative analysis, we only included one strain in the cases of yeasts carrying the same variant. According to our data, each killer-producing yeast harbors a different type of L-A with a degree of nucleotide variation that ranges from about 90% identity (in several *S. paradoxus* strains from close geographical location though producing different toxins, for example Sp T21.4 and Sp S28), to only about 73% identity (*S. paradoxus* N-45 strain from the Far Eastern collection, or the *S. kudriavzevii* viruses fall into this category) (Figure S2). With respect to the encoded Gag-pol proteins, they show amino acid (aa) identities that range from 85% to more than 98%. A dendrogram obtained using the Gag-Pol aa sequences that also includes the three known *S. cerevisiae* L-A helper variants is shown in Figure 2B. The tuber aestivum (Tav-1) totivirus was used as outgroup. There are features worth of noting. We observed a higher conservation in viruses from strains collected in proximal geographical locations (e.g., *S. paradoxus* strains from European collections are grouped into two clusters, indicated as E-1 and E-2), while *S. paradoxus* N-45 from Far East carries a quite different L-A, suggesting that this host may have been isolated from European populations for a long period of time, and thus the L-A it carries has a different evolutionary history. With respect to the L-A in *S. uvarum*, it is more closely related to the *S. paradoxus* European L-A variants. Interestingly, in the case of *S. kudriavzevii*, the L-A from FM1183 (a European strain) is quite separated from the L-A in IFO1802 (a Japanese strain). Thus, it appears that in the wild (under no pressure by domestication such as in *S. cerevisiae*) strains that are geographically proximal carry similar L-As, with independence to the type of toxin they may produce. Regarding to *S. cerevisiae*, L-A-lus and L-A-2 (present in wine strains) are closer, as already reported [18], while L-A (from K1 strains) is quite apart. Indeed laboratory strain Sc SK1 is probably the result of a recent cross between a West African strain and a European strain [33].

### 2.3. Killer Viruses Cross-Transmission between *Saccharomyces Sensu Stricto* Species

#### 2.3.1. Maintenance of *S. paradoxus* Viruses in *S. cerevisiae*

**Figure 3:**
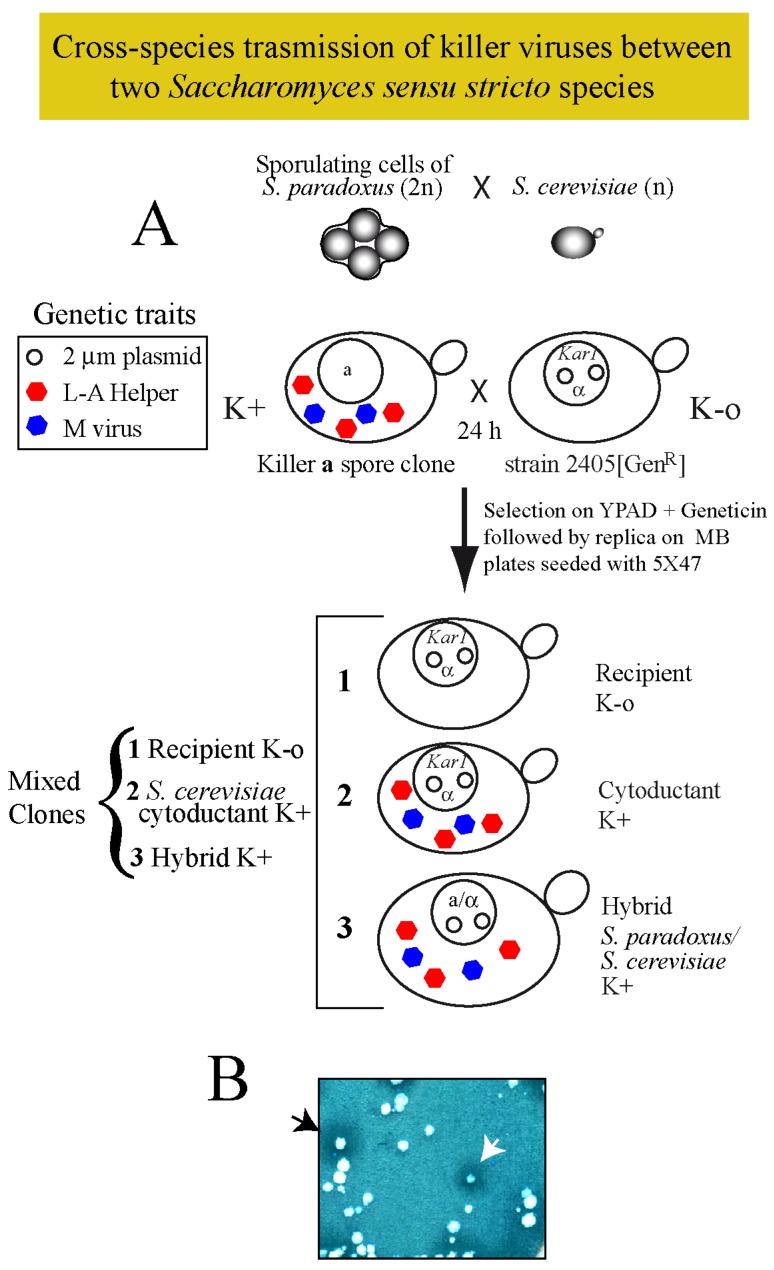
(**A**) Diagram of the experimental approach followed to introduce killer viruses from sporulating cells of a *S. paradoxus* killer strain into the haploid *S. cerevisiae* 2405 K-o strain carrying the *kar1* mutation and expressing the geneticin resistance marker from a vector (open circles in the nucleus). After growing both strains together in a rich medium agar-plate for 24 h, cells were streaked for single colony isolation on a plate with geneticin. Three types of cells can grow: (1) the original recipient K-o cells; (2) cytoductants that carry the nucleus of 2405 but they are killers (K+); and (3) hybrid diploids produced by mating *S. paradoxus* and *S. cerevisiae*, which are also killers (K+). (**B**) The panel shows an example of the three types of clones after replica plating on an MB plate seeded with the sensitive strain 5×47. Halos of growth inhibition surrounding certain colonies indicate successful transmission of viruses. The black arrowhead indicates a hybrid clone and the white arrowhead indicates a cytoductant (*S. cerevisiae* cells carrying viruses from *S. paradoxus*).

Purified virions from one species within the *sensu stricto* have been successfully introduced into other species of the same cluster in the lab [9,38] suggesting that there is no impairment for stable amplification in the new host. In various cases, however, from unknown reasons, results were negative. In addition, some of the data reported about transfection efficiency or about the type of viruses used were incorrect [38]. Thus, we followed a different approach to know whether cross-species transmission could happen in the wild. We focused our attention on *S. paradoxus* and *S. cerevisiae*, and asked if their killer viruses could be directly transmitted horizontally among them, by mimicking in the lab conditions found in the wild. We reasoned that given conditions of close proximity, germinating spores (or rare haploid cells) from one virus-carrying strain may mate to a compatible K-o germinating spore (or haploid cell) of another species, producing either killer hybrids or to a lesser extent, killer haploids if defects in karyogamy arose. Following this principle, we decided to introduce viruses from four *S. paradoxus* strains (S28, T21.4, N-45 or Q74.4) into *S. cerevisiae*. This was done by cytoplasmic mixing using as donor strains sporulating cultures of wild *S. paradoxus* diploids, and as the recipient one, *S. cerevisiae* strain 2405 expressing geneticin resistance (Figure 3A shows a schematic diagram of the procedure, as described in Methods).

**Figure 4:**
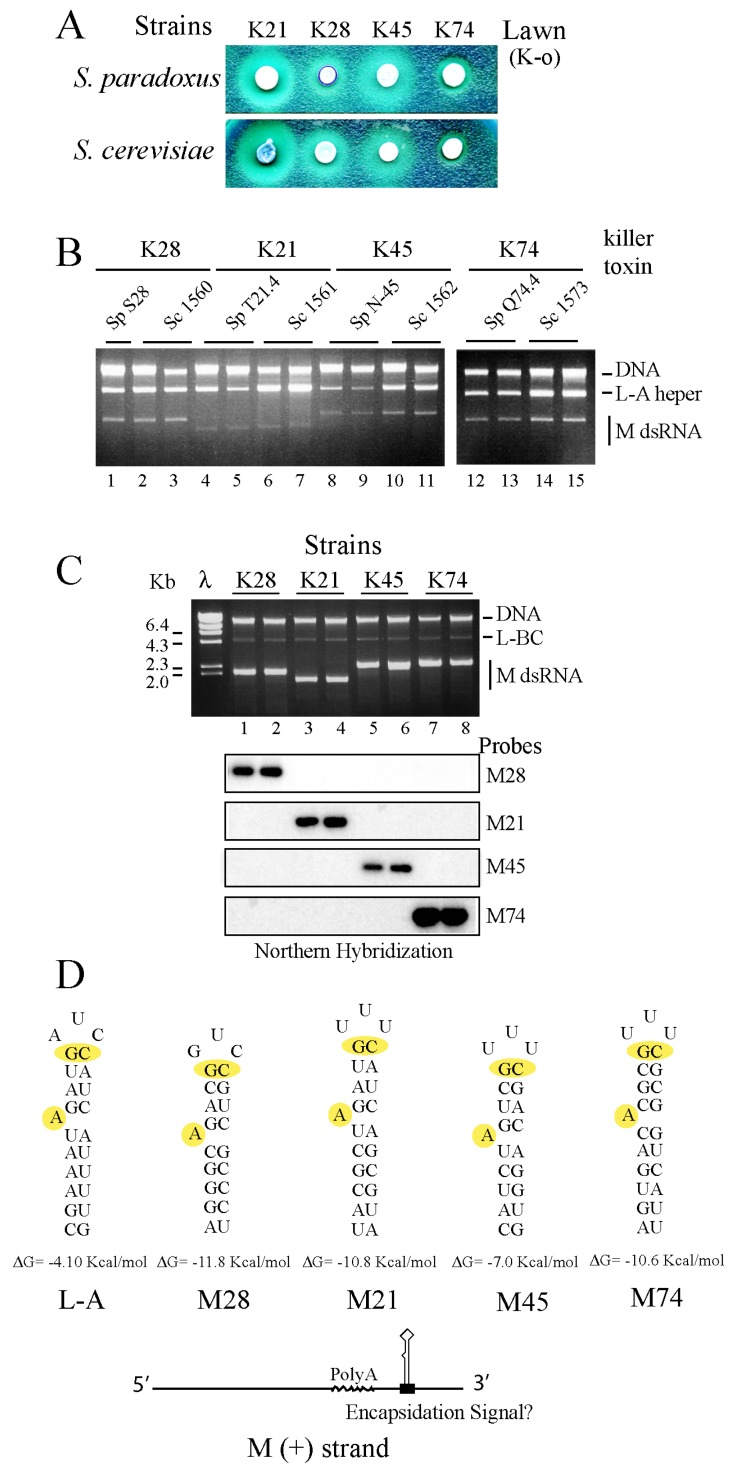
(**A**) Four *S. paradoxus* strains (upper panel) and their diploid *S. cerevisiae* derivatives (lower panel) were tested for killer activity on a lawn of the K-o strain 5×47. Similar amounts of each killer strain (ca. 5 × 10^5^ cells) were spotted. The size and aspect of the halos vary among the strains. (**B**) Two independent colonies from each strain (with the exception of S28 with only one) were grown for 2 days in rich media and RNA was extracted. After treatment with RNase A in the presence of 0.5 M NaCl the remaining nucleic acids (dsRNAs and DNA) were separated on an agarose gel and stained with ethidium bromide. The sizes of M dsRNAs vary among the strains. (**C**) M dsRNAs producing different killer toxins in *S. paradoxus* are maintained in *S. cerevisiae* by L-A coat proteins (different from their original helpers) expressed from a vector. The L-A coats exclude the helper viruses present in the donor strains. The minor dsRNA band with a size of 4.6 Kb is L-BC dsRNA, the genome of another totivirus in *S. cerevisiae* unrelated with the killer phenotype. Note that the amounts of each M dsRNA in these strains are at least 20-fold higher than those in *S. paradoxus* (**B**). After being transferred to a nylon membrane, dsRNAs were hybridized with four specific probes that recognized M28, M21, M45 or M74, respectively. The autoradiograms are shown below the ethidium bromide-stained gel. (**D**) All the M dsRNAs carry in their 3′ non-coding regions putative encapsidation signals similar to that of L-A. A prediction of their secondary structures by MFOLD is depicted. In yellow color, the two features in the signals necessary and sufficient for encapsidation, the protruding A and the GC pair close to the loop are shown. Below appears a diagram of M (+) strands showing the polyA internal region (wavy line) and the location of the putative encapsidation signals.

The recipient strain was *ρ*^+^, since our first tries with *ρ*° cells were unsuccessful (see below for explanations). We got in all cases several killer colonies that corresponded mostly to hybrids between parental strains, and few colonies of the recipient *S. cerevisiae* strain but killer. Both populations were distinguished by their different growth rate, auxotrophic markers, mating type and/or the capacity to sporulate (Figure 3B). In this way, we constructed strains 1530 (K28), 1531 (K21), 1546 (K45) and 1539 (K74), respectively (Table 1), all isogenic to 2405 but producing different killer toxins. To compare the killing capacity of *S. paradoxus* versus *S. cerevisiae*, we constructed for each toxin-producing haploid the corresponding diploid by crossing it to the K-o strain 2928. In this way, we obtained strains 1560 (K28), 1561 (K21), 1562 (K45) and 1573 (K74) (Table 1). Figure 4B shows a preparation of total nucleic acids from these two sets of *S. cerevisiae* or *S. paradoxus* diploid strains. We found equivalent amounts of the killer genomes (M and L-A dsRNAs) in these two species, with perhaps a minor increase in *S. cerevisiae*. When toxin activity was tested on a bioassay (Figure 4A) it seems that in some cases *S. cerevisiae* produces slightly bigger killing halos, but it is not a general trait for all toxins. Bigger killing halos may reflect different amounts of toxins or a better processing or secretion of the toxins (or both). Differences, however, are minor.

#### 2.3.2. Killer Viruses Cross-Transmission between Other *Saccharomyces sensu stricto* Species

We also successfully introduced K1 or K2 viruses from haploid *S. cerevisiae* strains (2403 or 1137, respectively) into sporulating cells of *S. kudriavzevii* IFO1082 strain as described in Methods, obtaining either hybrids (*S. cerevisiae/S. kudriavzevii*) or *S. kudriavzevii* diploid strains that could be distinguished by ITS analysis [49]. The diploid cytoductants were originally haploids, but as the recipient cells were homothallic, some of them became stable diploids through spontaneous diploidization due to mating type switches (confirmed by the capacity to sporulate of the latter cells). Sk IFO1082 recipient cells carried a helper virus distinct from L-A of the K1-donor *S. cerevisiae* cells (Figure S2). RT-PCR amplification followed by sequencing, and Northern analysis with an L-A-specific probe showed that the original L-A variant in Sk IFO1082 had been displaced by the incoming L-A (Figure S3A) indicating an exclusion phenomenon between both helpers. It also suggests that the L-A variant in *S. kudriavzevii* cannot maintain M1 dsRNA, since our selection procedure was based on the transmission of the K1 killer phenotype. Finally, K1 or K2 viruses from strain Sc 1332 or Sc 1337, respectively, were transferred into *S. uvarum*; L-A-lus alone from strain Sc 1333 was also introduced into the same recipient yeast. Similar to previous cases, we used *ρ*^+^ recipient cells due to incompatibility problems between nuclei from *S. uvarum* and mitochondria from *S. cerevisiae* (see below). All the *S. cerevisiae* dsRNA viruses were stably maintained in the new foster species after more than 100 generations.

In summary, our data indicate that at least seven different killer viruses from two species (*S. paradoxus* and *S. cerevisiae*) can be stably maintained in all the members of the *sensu stricto* genus so far analyzed (four) and with similar amounts of virions, when compared the donor and the recipient strains.

### 2.4. Mitochondria Incompatibility as the Main Barrier for Killer Viruses Spreading in the Wild?

**Figure 5:**
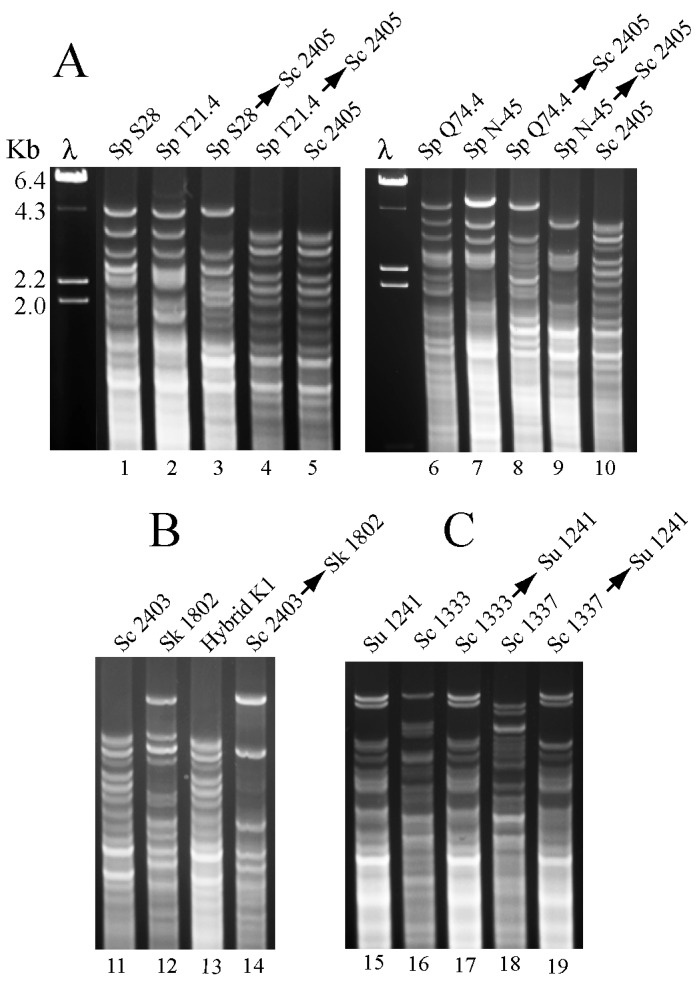
(**A**) RFLP mitochondrial analysis of *S. paradoxus* and their *S. cerevisiae* derivative killer strains. Viruses from four *S. paradoxus* (Sp) strains: S28 (lane 1), T21.4 (lane 2), Q74.4 (lane 6), or N-45 (lane 7) were introduced by cytoduction into *S. cerevisiae* (Sc) strain 2405 (lanes 5 and 10). Cytoductants are indicated by two names separated by an arrow (the first name is the donor and the second the recipient). DNA digested with HinfI was separated in agarose gels and stained with Ethidium bromide. λ, lambda DNA digested with HindIII used as mobility markers. (**B**) A similar analysis was done with *S. kudriavzevii* (Sk) strain 1802 (lane 12) and a cytoductant that received K1 viruses from Sc strain 2403 (lane 14). In the gel, we also show the mitochondrial pattern of one hybrid diploid strain between Sk and Sc parental strains (lane 13). (**C**) L-A-lus or L-A-2 viruses were cytoduced from Sc strains 1333 or 1337, respectively, into *S. uvarum* (Su) haploid strain 1241.

As mentioned above, our first attempts to cytoduce killer viruses from *S. paradoxus* into *S. cerevisiae ρ*° cells were unsuccessful. We were unable to recover *S. cerevisiae* killer cells that were also respiratory proficient. This could be due to two different reasons: (1) killer viruses from *S. paradoxus* were not stable in *S. cerevisiae*; or (2) mitochondria from *S. paradoxus* were not functional in *S. cerevisiae*. A search in the literature indicated that the latter was likely to be the case, since only few *S. paradoxus* strains have been shown to carry mitochondria able to complement the respiratory deficiency in *S. cerevisiae* [50]. We solved this problem using recipient cells that were *ρ*^+^. During the mating process, mitochondria of both parents were present, and since nuclei of *S. cerevisiae* appear to be incompatible with mitochondria of *S. paradoxus*, our cytoductants were presumed to carry always the mitochondria of the recipient. However, we frequently observed killer cytoductants with slightly different growth rates, or even cases in which the cytoductants were *ρ*°. Thus, we decided to analyze their mitochondria by performing Restriction Fragment Length Polymorphism (RFLP) analysis as described [51]. While some killer cytoductants had the same mitochondrial DNA (mtDNA) restriction pattern as the recipient (Figure 5A, see lanes 4 and 5), others showed patterns different from either parental strain (lanes 3, 8 or 9) suggesting the existence of recombination between both parental mtDNAs. None of the newly constructed strains carried *S. paradoxus* mitochondria, confirming that they are not functional in *S. cerevisiae*. We also analyzed few of the hybrids obtained, and their mitochondria appeared mostly derived from *S. paradoxus* (not shown). A similar RFLP analysis was performed in cross-transmission experiments of killer viruses from *S. cerevisiae* into *S. kudriavzevii* (Figure 5B) or into *S. uvarum* (Figure 5C). Again, none of the cytoductants carries the donor mitochondria. They have either the recipient ones (all *S. uvarum* cytoductants) or hybrid mitochondria (the *S. kudriavzevii* cytoductant).

It is worth noting that, in all cross-species transmission experiments, hybrid diploids accounted for most of the killers, and that they were obtained at high frequency. Hybrids between *S. paradoxus* and *S. cerevisiae* can be easily obtained in the lab [52]; however, there are not so many reports of natural hybrids between these two species [53,54], probably reflecting the fact that *S. paradoxus* exists mainly in the wild, while *S. cerevisiae* lives mostly in domesticated environments. From our data, we believe that horizontal cross-transmission of killer viruses in the wild may not be a difficult task, provided the proximity of cells from both species, and that mitochondrial incompatibility may represent the main obstacle for this to occur thus compromising the spreading of these viruses and the toxins they produce in the wild.

### 2.5. M28, M21, M45 and M74 Can Be Maintained by L-A Proteins Expressed from a Vector

Works by Schmitt and coworkers have shown that M28 virions from strain S28 when introduced into *S. cerevisiae* by transfection could be maintained by their helper virus (L-A-28) or by the resident L-A virus [9]. We confirmed the latter case when viruses from the K28 strain 1530 were introduced into *S. cerevisiae* strain 2928 expressing Gag and Gag-Pol of L-A from a vector [55]. The cytoductants were stable K28 killers and Northern hybridization showed that the L-A coat proteins expressed from the plasmid competed with L-A-28 and eliminated it (Figure 4C). Following a similar approach, we also constructed K21-, K45- or K74-producing cells, all of them supported by L-A coat proteins expressed from the same vector (Figure 4C) and without their original helper viruses. As expected, the amounts of the M dsRNAs are greatly increased (more than 20-fold), as compared to the original *S. paradoxus* producers or to the *S. cerevisiae* diploids, which maintain M viruses by their specific helpers (Figure 4B). Concomitantly, their killer activity is also increased (not shown). Thus, we have constructed a collection of “superkiller” *S. cerevisiae* strains quite useful for further characterization of the M dsRNAs (or the toxins they produce). The increased M amounts have facilitated their purification for sequencing purposes. Toxin overproduction will be also a great advantage for future characterization of their mode of action. All these M dsRNAs have the same structure found in other pptox-encoding M dsRNAs, that is an ORF in the 5′ region followed by an internal PolyA sequence and a 3′ non-coding region (Figure 4D). Interestingly, in the 3′ regions, we found nucleotide stretches (one or two) predicted to fold into a secondary stem-loop structure similar to that of the encapsidation signal of L-A. Thus, these structures seem to be good candidates to work as the encapsidation signals recognized by the L-A RNA polymerase expressed from the vector. The existence of these sequences, with a strict conservation of two features essential for encapsidation (shown in yellow color in Figure 4D) may explain why all these M dsRNAs from *S. paradoxus* can be maintained stably by L-A coats (when over-expressed from a vector), a helper substantially different from the one they are dependent on in their natural host.

## 3. Discussion

### 3.1. Variation of L-A Helper Sequences Depend Mostly on the Geographical Location of the Host

In this work, we have characterized killer viruses from different *Saccharomyces sensu stricto* species, namely *S. paradoxus*, and clarified previous reports that wrongly assigned the production of certain killer toxins to *S. cerevisiae* and/or to *S. paradoxus*. Of special importance was the analysis of K1 toxin producers, since there are reports analyzing the possible co-evolution of K1 virions and their hosts. Virions had been purified from different K1 killer strains and later on introduced into new hosts under controlled lab conditions [38]. Since the initial characterization was not done properly, the conclusions drawn have to be taken with caution. We used the same strains reported in those studies, and our data show that the only methodology that assigns without doubt a particular toxin to one species is based on its molecular characterization, since each toxin is encoded by a specific M dsRNA with a unique nucleotide sequence. The bioassay methodology based on the capacity of killing (or not) reference strains producing known killer toxins is not always reliable, since some of the strains used may be naturally resistant to the new toxins, or simply the new toxins may not be active enough in the conditions used. RT-PCR analysis can also lead to wrong assignments, since cross-contamination in the bench may occur when working with different RNA preparations simultaneously. Thus, we emphasize the importance of Northern analysis with specific probes (Figure 1A) before further sequencing. Our data indicate that at least five different toxins are produced in *S. paradoxus* and three in *S. cerevisiae* and none of them is secreted by strains from both species. This characterization was needed before analyzing the helper viruses maintaining them. We had observed in the case of *S. cerevisiae* Klus and K2 viruses from wine strains that each M satellite was maintained by a specific helper virus with about 75% of their nt identical, and wanted to extend our analysis to other killer toxin-producing viruses. Our data of nine new L-A variants (some associated to specific M killer viruses and some coming from non-killer strains) show that these helpers have nucleotide sequences with a maximum variation that reach up to 28% in the more divergent cases and is as low as 8% in cases when the helpers support M satellites producing similar toxins (e.g., the three cases of M28-type viruses in *S. paradoxus* strains S28, DBVPG5640 or CECT1939, Figure 2B, fall into this category). Of particular interest, however, are those cases of yeast strains that producing different toxins carry L-A variants quite close evolutionarily (Figure 2B). They have in common a proximal geographical location of their hosts, coming from ecological niches that frequently overlap. This may have facilitated the horizontal transmission of the L-A helpers among them, not only within the same species, but also among distinct species. For example, viruses found in *S. uvarum* are more closely related to some *S. paradoxus* viruses, than viruses found in *S. paradoxus* strains isolated from far locations (the latter case is that of strain N-45 from the far East, that carries a variant quite different from any L-A variant found in *S. paradoxus* European strains). A similar situation is found in the two *S. kudriavzevii* strains analyzed (both non-killer). Apparently, their geographical isolation led to a drastic drift in their nucleotide sequences. The similarity found in viruses from hosts of proximal geographical locations, also suggests the existence of horizontal cross-species transmission in the wild. In the lab, and by mimicking conditions of close proximity, we found no impairment for this transmission to occur, and also for the stable amplification of these killer viruses in all new hosts so far analyzed.

### 3.2. Host–Virus Adaptation Previous to Speciation

The lack of host specificity within the *sensu stricto* species observed in our experimental data, is in contrast to reports about host–virus co-adaption among the different species and the viruses they carry, claimed by some authors [38,56]. Our conclusions are based on the following: (1) In the same species, various killer viruses can be found (three in *S. cerevisiae* and five in *S. paradoxus*), and the amounts of virions each strain carry depends more on the type of virus (K1, K2, K28, etc.) than on the combination “certain host–certain virus”. For example, in general M1 and its helper virus L-A are present in higher amounts as compared to M2 or Mlus in *S. cerevisiae.* In fact, when K1 viruses were introduced into *S. uvarum*, or *S. kudriavzevii*, these two foster species also maintained higher amounts of K1 viruses. (2) When introduced into other species, the amounts of viruses each recipient species can maintain are quite similar to the ones in the donor strains, as shown here (Figure 4A,B). Differences observed in their killing capacity may be more correlated to the toxin production itself in the new host (processing, secretion, etc.) than to an adaptation between the foster host and the virus. In fact, these viruses seem to function more like a well-adapted “organelle” due to the absence of an extracellular route of transmission. (3) The lack of host specificity also means that, in the different species, the viruses use the same (or very similar) strategies to evade host surveillance mechanisms. One of them is being able to escape the cytoplasmic Xrn1p 5′ exonuclease conserved in all *sensu stricto* species. Contrary to reported [56], we did not observe any correlation between a host specific Xrn1p and the type of virus the host carries. In fact, we have already shown that over-expression of Xrn1p from *S. cerevisiae* has different effects on K1, K2 or Klus viruses (all of them from the same species) [17]. Our unpublished data [44] also suggest that M21, M45 or M28 viruses (all of them from *S. paradoxus*), have similar decrease in their copy numbers after *S. cerevisiae* Xrn1p over-expression. (4) The viruses share common pathways to use host metabolism in their own benefit. One example is the cap-snatching mechanism characterized in the K1 killer viruses, which provides cap groups to the 5′ ends of the viral transcripts from host mRNAs [57]. With the set of strains constructed here that maintained different *S. paradoxus* killer viruses by L-A proteins produced by a vector we have confirmed that this mechanism applies to all M satellites. A mutant plasmid expressing a Gag protein altered in the His154 residue needed for cap-snatching produces either non-killer or weak killers, although they can maintain high amounts of virions [44].

The adaptation between the yeast host and the L-A totivirus within the *sensu stricto* cluster (that obviously should have existed to originate the killer strains we observe nowadays) probably had occurred before the separation and genetic isolation that have produced the seven species currently assigned to the taxon (see Introduction). This would mean that viruses in all these species have the same (or almost the same) metabolic requirements. It also suggests that the presence of viruses in all of them apparently does not affect substantially the host metabolism, either because they accumulate in conditions in which normal growth has stopped (stationary phase cells) and thus the price of maintaining the viruses is minimized when energy sources or factors are no longer required by the host, or because those requirements are minimal. In fact, recent reports indicate low to moderate changes in host gene expression levels in response to the loss of killer viruses [58,59].

### 3.3. Origins of Toxin-Encoding M Satellites

The structures of the M satellites are similar in all cases analyzed. The existence of internal PolyA regions of about 200 nt in length is reminiscent of the 3′ terminal polyA of host mRNAs, suggesting that they originated from ORFs encoded in the yeast genome. As mentioned, some of those ORFs are still present in the *sensu stricto* cluster or other related yeast genomes, either as frames encoding proteins with putative toxin activity or in some cases with only parts of the ORFs remaining. Does it mean that once captured by the helper viruses the resident ORFs were no longer needed and thus mutations accumulated without any selective pressure such that most of the original information was lost? If that is the case, it also means that the killer toxins we see nowadays in viruses have an ancient origin and the M viruses encoding them share a long history in the yeast cells with their helpers, originating hosts that outcompete other non-toxin-producing strains. Only few ORFs with toxin activity have been identified in each yeast genome, but with only one copy per genome, it is possible that some putative toxins are not expressed high enough to produce a killer phenotype. When expressed from a virus, there is a strong amplification (hundreds of copies per cell), and thus toxin production is increased over the detection limits in the standard bioassays of killer activity.

We observed in the M dsRNAs 3′ non-coding regions essential *cis* signals for replication and encapsidation. One very well conserved signal is the stem-loop structure involved in encapsidation (one or two per molecule, Figure 4D). There are intriguing questions about the conservation of these signals. (1) Are they the result of independent recombination events for each toxin-encoding RNA and an ancestral L-A virus? (2) Is each toxin-encoding satellite derived from a previous one by substituting the pptox region from a new one? [22]. This would be followed by further changes in the non-conding regions nucleotide composition due to lack of proofreading of the viral RNA-dependent RNA polymerase. We believe that the latter is likely to occur, and it may explain why in controlled lab conditions the same L-A coat proteins can support different M satellites, as shown here (Figure 4C). With respect to the evolutionary history of each M dsRNA, we think that differences between the nucleotide (or amino acid) sequences of the host ORFs and the pptox reflect the time passed from the jumping event (the moment the ORF was first expressed from the virus) and the present time. Those cases where host information is lost may be more ancients than cases in which a strong similarity still remains.

## 4. Materials and Methods

### 4.1. Yeast Strains and Media

Strains used are described in Table 1. Wild strains are prototrophic and homothallic. Media and incubation conditions were as described [17].

### 4.2. Cytoduction (Cytoplasm Mixing)

Cytoduction is a mating procedure used to transfer cytoplasmic elements such as dsRNA viruses or mitochondria from a donor to a recipient strain without nuclear fusion, as one of the strains involved is a *kar1* mutant defective in karyogamy [60]. Usually the recipient strain is respiratory deficient (*ρ*°) and the cytoductants are selected in glycerol-containing plates (*ρ*^+^). In this work, we used slightly different conditions: i.e., when one of the strains involved was a prototroph diploid, the strain was induced to sporulate before mating. Three types of virus cross-transmission experiments were performed: (1) To transfer killer viruses from *S. paradoxus* into *S. cerevisiae* we used as donor strains sporulating cultures of wild *S. paradoxus* strains (S28, T21.4, Q74.4 or N-45, Table 1) and as the recipient strain *S. cerevisiae* 2405 (*kar1* mutant) carrying a geneticin resistance marker. Our initial attempts were unsuccessful, since there is a strong incompatibility between *S. cerevisiae* nucleus and *S. paradoxus* mitochondria [61]; thus, in successive experiments the recipient strain 2405 was *ρ*^+^. After mating for 24 h on rich agar medium (YPAD), the donor cells were eliminated by single colony isolation on YPAD plates supplemented with 200 μg/mL of geneticin and the cytoductants were selected by their killer activity; histidine auxotrophy was used to differentiate between the cytoductants and some *S. cerevisiae/S. paradoxus* hybrids. (2) K1 or K2 killer viruses were also transferred from *S. cerevisiae* strains 2403 or 1137, respectively, to *S. kudriavzevii* wild strain IFO1082. In this case, killer cells were mixed with a sporulating population of *S. kudriavzevii* as above, followed by single colony isolation on—his plates (to eliminate the donor strains). Killer colonies were then selected and further characterized to differentiate between hybrids or cytoductants. (3) The introduction of K1, K2 or Klus viruses from *S. cerevisiae* into *S. uvarum* strain 1241 was done following the standard procedure, since in this case all the strains used were laboratory heterothallic haploids with different auxotrophic markers that enable the selection of cytoductants. Again the *S. uvarum* strain was *ρ*^+^ since mitochondria from *S. cerevisiae* are not functional in *S. uvarum*. Once we obtained *S. cerevisiae* strains carrying different *S. paradoxus* viruses (see results) the viruses could be transferred to other *S. cerevisiae* haploid heterothallic strains by standard cytoduction procedures.

### 4.3. Killer Assay

Killer activity was tested using MB plates seeded with a lawn of the sensitive strain 5 × 47 or different killer strains. Isolated colonies of the strains to be assayed were replica plated on the MB plates. Alternatively, 2 μL drops of overnight liquid cultures (5 × 10^5^ cells) were directly spotted on the MB plates. The incubation temperature was 20–22 °C and the killer toxin activity was indicated by a halo of growth inhibition of the sensitive tester strain.

### 4.4. cDNA Synthesis and Sequencing of L-A Variants from *Saccharomyces sensu stricto*

First, we amplified a 2.2-Kb fragment spanning the frame shifting region and encapsidation signal by RT-PCR using oligos NR121 and NR122, as previously done for L-A variants in *S. cerevisiae* [18]. To analyze the remaining regions (the most 3′ end region and the 5′ half that contains the Gag domain, Figure 2A), we used other three oligos (NR138 5′-AATTAGAGCATATGGGTA-3′, NR139 5′-AACTCCCCATGCTTAGAT-3′ and NR140 5′-TTCTCCGGTAGGCATTAC-3′) for RT-PCR amplification (Figure 2A). NR138 annealed to the last 11 nt of all the L-A variants so far analyzed and was used in combination with internal oligos complementary to sequences close to the 3′ end of the 2.2-Kb fragment. With respect to the 5′ half, oligo NR139 anneals to the AUG initiation codon region for Gag and oligo NR140 in the frame shifting region, both highly conserved in *S. cerevisiae* L-A variants. The nucleotide sequences of the amplified regions were obtained using the mentioned oligos, along with other internal primers designed after parts of the sequences had been established.

### 4.5. Northern Hybridization and Plasmids

Total nucleic acids were obtained from 1 mL stationary-phase cultures by breaking cells with glass beads followed by phenol extraction and ethanol precipitation as described [62]. Total RNAs were separated on native agarose gels, denatured by treatment with formamide and formaldehyde and transferred to neutral nylon membranes [63]. In certain experiments, before the separation in the gel, samples were first digested with RNase A (10 μg/mL) at 37 °C for 12 min in the presence of 0.5 M NaCl, followed by phenol/chloroform/isoamylic alcohol extraction and ethanol precipitation. The treatment produces the selective digestion of single-stranded RNA, whereas the dsRNA is resistant. RNAs were detected with ^32^P-labeled-specific probes made by T3 or T7 run-off transcription from plasmids previously digested with restriction enzymes to render them linear. Plasmids used to prepare probes to detect L-A and M1 were described elsewhere [17]. The M28 probe was synthesized from plasmid pRE1469, which contains a fragment of 547-bp of M28 cDNA (from nt 13 to nt 559) obtained by RT-PCR. The fragment was cloned between the EcoRI-SpeI sites of Bluescript KS+ vector. Plasmids pRE1476, pRE1477 or pRE1478 were used to make specific probes for M21, M45 or M74 dsRNAs, respectively. The cDNA fragments were cloned into the unique BamHI site of the Bluescript KS+ vector. pRE1476 contains a 542-bp cDNA fragment from M21 (from nt 7 to nt 549), pRE1477 contains a 651-bp cDNA fragment from M45 (from nt 7 to nt 657) and pRE1478 contains a 620-bp cDNA fragment from M74 (from nt 142 to nt 761). The L-A-28 probe was made from plasmid pRE1467 that contains a 1250-bp cDNA fragment from L-A-28 (from nt 2726 to nt 3975) cloned between the XhoI and BamHI sites of Bluescript KS+ vector. pI2L2 is a 2 μm-based plasmid which over-expresses L-A coat proteins under the constitutive *PGK1* promoter [55]. It can maintain M1 or M2 dsRNA satellites in the absence of their helper viruses (L-A or L-A-2, respectively) [18,55]. Plasmid pRE1334 is a 2 μm-derivative multicopy plasmid that provides geneticin resistance (Gen^R^). It was used to distinguish donor and recipient strains in certain cytoduction experiments when no auxotrophic markers where available. In the absence of selective pressure, this plasmid is eliminated easily from the cells.

### 4.6. Other Procedures

Enzyme digestions and cloning procedures were done according to standard methods [64]. Plasmid DNA was obtained using Wizard Plus SV Minipreps DNA Purification System (Promega, Madison, WI, USA). DNA fragments for cloning and sequencing were purified with QIAquick gel extraction kit (Qiagen, Hilden, Germany). RFLP analysis of mitochondrial DNA (mtDNA) was performed according to [51]. PCR amplification of the 5.8S-ITS rDNA for yeast identification was done according to [49]. RNA secondary structure predictions were done using the MFOLD program [65].

### 4.7. GenBank Accession Numbers

Nucleotide sequence accession numbers for the new L-A variants are KY489962 (*S. paradoxus* virus L-A-T21.4), KY489963 (*S. paradoxus* virus L-A-N45), KY489964 (*S. paradoxus* virus L-A-Q74.4), KY489965 (*S. paradoxus* virus L-A-DBVPG4650), KY489966 (*S. paradoxus* virus L-A-CECT1939), KY489967 (*S. paradoxus* virus L-A-CECT11143), KY489968 (*S. paradoxus* virus L-A-Q62.5), KY489969 (*S. uvarum* virus L-A-CECT10560) and KY489970 (*S. kudriavzevii* virus L-A-IFO1082). The accession number for M28 ORF from *S. paradoxus* strain CECT1939 (CBS432) is MF358735. Accession numbers for the complete preprotoxin-encoding M dsRNAs in *S. paradoxus* strains are: MF358732 (M21 from Sp T21.4), MF358733 (M45 from Sp N-45), MF358734 (M74 from Sp Q74.4) and MG011653 (M62 from Sp Q62.5). The sequences were obtained by High-throughput sequencing (HTS) technology by Illumina with purified dsRNAs, and were assembled using A5-miseq v. 20160825. Sequences at the ends were confirmed by 3′-RACE.

## Figures and Tables

**Table 1:** Yeast strains used.

Strain	Description and or Genotype
***S. paradoxus* (Sp)**	
S28	Europe (wine); K28
T21.4	United Kingdom (oak trees); K21
Q74.4	United Kingdom (oak trees); K74
DBVPG4650	Italy (fossilized guano); K28
Q62.5	United Kingdom (oak trees); K62
Y8.5	United Kingdom (oak trees); K74
CECT11143/(CBS5829)	Denmark (moor soil); K-o
CECT1939/(CBS432)	Russia (oak trees); K28
N-45	Far East (mongolian oak); K45
***S. uvarum* (Su)**	
CECT10560	Spain (wine)
CECT1884	Spain (wine)
1241	*MAT*α *ho∆*::*NatMX his3 lys2 ura3*
***S. Kudriavzevii* (Sk)**	
CECT11825/(IFO1802)	Japan (decayed leaf)
***S. cerevisiae* (Sc)**	
SK1	Standard laboratory K1 killer strain
NCYC 232	Ex-American Yeast Foam
5X47	Diploid tester strain sensitive for killer assay
2928	α *ura3 his3 trp1* L-A-o
2405	α *his4-1, kar1-1* L-A-o
2403	α *his4-1, kar1-1* L-A, M1
1137	α *his4-1 kar1-1*, L-A-2, M2
1332	α *leu1, trp1, kar1-1* L-A, M1
1333	α *leu1, trp1, kar1-1* L-A-lus
1337	α *leu1, trp1, kar1-1* L-A-2, M2
1530	2405 with L-A-28 and M28 from Sp S28 (obtained by cytoduction)
1531	2405 with L-A-T21.4 and M21 from Sp T21.4 (obtained by cytoduction)
1539	2405 with L-A-Q74.4 and M74 from Sp Q74.4 (obtained by cytoduction)
1546	2405 with L-A-N45 and M45 from Sp N-45 (obtained by cytoduction)
1560	diploid between 2928 and 1530 (L-A-28 and M28 from Sp S28)
1561	diploid between 2928 and 1531 (L-A-T21.4 and M21 from Sp T21.4)
1562	diploid between 2928 and 1546 (L-A-N45 and M45 from Sp N-45)
1573	diploid between 2928 and 1539 (L-A Q74.4 and M74 from Sp Q74.4)

Unless otherwise indicated, laboratory strains were constructed in this work or previous work from our laboratory. Geographical origins of *S. paradoxus* strains (except for strain S28) were obtained from [33]. In the descriptions of strains obtained by cytoduction, the first strain is the recipient one and the second is the donor strain. *S. uvarum* strain 1241 is derived from strain JRY8150 provided by C. Hittinger (University of Wisconsin-Madison, USA). Strains 5X47, 2928, 2405 and 2403 are from R. B. Wickner’s laboratory (National Institutes of Health, USA).

## Supplementary Materials

The following are available online at www.mdpi.com/2072-6651/9/10/313/s1, Figure S1: Analysis of M dsRNAs from strains Sp Q62.5 and Sp Y8.5 by Northern hybridization, Figure S2: Nucleotide sequence identity of L-A variants obtained by a ClustalW comparison, Figure S3: (**A**) Detection of L-A and M1 in K1 killer cells of a *S. cerevisiae/S. kudriavzevii* hybrid diploid or *S. kudriavzevii* cytoductants. (**B**) 5.8S-ITS rDNA sequencing of *S. cerevisiae*, *S. kudriavzevii* or a hybrid diploid of both species, Table S1: Cross-killing activity of *Saccharomyces* killer strains.

## References

[1] Wickner R.B., Ghabrial S.A., Nibert M.L., Patterson J.L., Wang C.C., King A.M.Q., Adams M.J., Carstens E.B., Lefkowits E.J. (2011). Family Totiviridae. Virus Taxonomy: Classification and Nomenclature of Viruses: Ninth Report of the International Committee on Taxonomy of Viruses.

[2] Esteban R., Fujimura T., Wickner R.B. (1989). Internal and terminal cis-acting sites are necessary for in vitro replication of the L-A double-stranded RNA virus of yeast. EMBO J..

[3] Icho T., Wickner R.B. (1989). The double-stranded RNA genome of yeast virus L-A encodes its own putative RNA polymerase by fusing two open reading frames. J. Biol. Chem..

[4] Dinman J.D., Icho T., Wickner R.B. (1991). A -1 ribosomal frame shift in a double-stranded RNA virus of yeast forms a gag–pol fusion protein. Proc. Natl. Acad. Sci. USA.

[5] Schmitt M.J., Breinig F. (2006). Yeast viral killer toxins: Lethality and self-protection. Nat. Rev. Microbiol..

[6] Wickner R.B., Fujimura T., Esteban R. (2013). Viruses and prions of *Saccharomyces cerevisiae*. Adv. Virus Res..

[7] Bevan E.A., Herring A.J., Mitchell D.J. (1973). Preliminary characterization of two species of dsRNA in yeast and their relationship to the “killer” character. Nature.

[8] Hannig E.M., Leibowitz M.J. (1985). Structure and expression of the M2 genomic segment of a type 2 killer virus of yeast. Nucleic Acids Res..

[9] Schmitt M.J., Tipper D.J. (1990). K28, a unique double-stranded RNA killer virus of *Saccharomyces cerevisiae*. Mol. Cell. Biol..

[10] Rodríguez-Cousiño N., Maqueda M., Ambrona J., Zamora E., Esteban R., Ramírez M. (2011). A new wine *Saccharomyces cerevisiae* killer toxin (Klus), encoded by a double-stranded RNA virus, with broad antifungal activity is evolutionarily related to a chromosomal host gene. Appl. Environ. Microbiol..

[11] De la Peña P., Barros F., Gascón S., Lazo P.S., Ramos S. (1981). Effect of yeast killer toxin on sensitive yeast cells of *Saccharomyces cerevisiae*. J. Biol. Chem..

[12] Martinac B., Zhu H., Kubalski A., Zhou X.L., Culbertson M., Bussey H., Kung C. (1990). Yeast K1 killer toxin forms ion channels in sensitive yeast spheroplasts and in artificial liposomes. Proc. Natl. Acad. Sci. USA.

[13] Lukša J., Podoliankaitė M., Vepštaitė I., Strazdaitė-Žielienė Ž., Urbonavičius J., Servienė E. (2015). Yeast β-1,6-glucan is a primary target for the *Saccharomyces cerevisiae* K2 toxin. Eukaryot Cell.

[14] Orentaite I., Poranen M.M., Oksanen H.M., Daugelavicius R., Bamford D.H. (2016). K2 killer toxin-induced physiological changes in the yeast *Saccharomyces cerevisiae*. FEMS Yeast Res..

[15] Eisfeld K., Riffer F., Mentges J., Schmitt M.J. (2000). Endocytotic uptake and retrograde transport of a virally encoded killer toxin in yeast. Mol. Microbiol..

[16] Schmitt M.J., Tipper D.J. (1995). Sequence of the M28 dsRNA: Preprotoxin is processed to an α/β heterodimeric protein. Virology.

[17] Rodríguez-Cousiño N., Gómez P., Esteban R. (2013). L-A-lus, a new variant of L-A totivirus in wine yeasts associated to Klus killer toxin-producing Mlus dsRNA. Possible role of satellite RNAs encoding killer toxins on the evolution of their helper viruses. Appl. Environ. Microbiol..

[18] Rodríguez-Cousiño N., Esteban R. (2017). Relationships and Evolution of Double-Stranded RNA Totiviruses of Yeasts Inferred from Analysis of L-A-2 and L-BC Variants in Wine Yeast Strain Populations. Appl. Environ. Microbiol..

[19] Konovalovas A., Serviené E., Serva S. (2016). Genome Sequence of *Saccharomyces cerevisiae* Double-Stranded RNA Virus L-A-28. Genome Announc..

[20] Park C.M., Bruenn J.A., Ganesa C., Flurkey W.F., Bozarth R.F., Koltin Y. (1994). Structure and heterologous expression of the *Ustilago maydis* viral toxin KP4. Mol. Microbiol..

[21] Schmitt M.J., Neuhausen F. (1994). Killer toxing-secreting double-stranded RNA mycoviruses in the yeasts *Hanseniaspora uvarum* and *Zygosaccharomyces bailii*. J. Virol..

[22] Ramírez M., Velázquez R., Maqueda M., López-Piñeiro A., Ribas J.C. (2015). A new wine *Torulaspora delbrueckii* killer strain with broad antifungal activity and its toxin-encoding double-stranded RNA virus. Front. Microbiol..

[23] Ivannikova Y.V., Naumova E.S., Naumov G.I. (2007). Viral dsRNA in the wine yeast *Saccharomyces bayanus var. uvarum*. Res. Microbiol..

[24] Pieczynska M., de Visser J.A.G.M., Korona R. (2013). Incidence of symbiotic dsRNA ‘killer’ viruses in wild and domesticated yeast. FEMS Yeast Res..

[25] Chang S.L., Leu J.Y., Chang T.H. (2015). A population study of killer viruses reveals different evolutionary histories of two closely related *Saccharomyces* sensu stricto yeasts. Mol. Ecol..

[26] Kurtzman C.P. (2003). Phylogenetic circumscription of *Saccharomyces*, *Kluyveromyces* and other members of the *Saccharomycetaceae*, and the proposal of the new genera *Lachancea*, *Nakaseomyces*, *Naumovia*, *Vanderwaltozyma* and *Zygotorulaspora*. FEMS Yeast Res..

[27] Kurtzman C.P., Robnett C.J. (2003). Phylogenetic relationships among yeasts of the ‘*Saccharomyces* complex’ determined from multigene sequence analyses. FEMS Yeast Res..

[28] Nguyen H.V., Gaillardin C. (2005). Evolutionary relationships between the former species *Saccharomyces uvarum* and the hybrids *Saccharomyces bayanus* and *Saccharomyces pastorianus*; reinstatement of *Saccharomyces uvarum* (Beijerinck) as a distinct species. FEMS Yeast Res..

[29] Naumov G.I., Naumova E.S., Masneuf I., Aigle M., Kondratieva V.I., Dubourdieu D. (2000). Natural polyploidization of some cultured yeast *Saccharomyces sensu stricto*: Auto- and allotetraploidy. Syst. Appl. Microbiol..

[30] Wang S.A., Bai F.Y. (2008). *Saccharomyces arboricolus* sp. nov., a yeast species from tree bark. Int. J. Syst. Evol. Microbiol..

[31] Libkind D., Hittinger C.T., Valério E., Gonçalves C., Dover J., Johnston M., Gonçalves P., Sampaio J.P. (2011). Microbe domestication and the identification of the wild genetic stock of lager-brewing yeast. Proc. Natl. Acad. Sci. USA.

[32] Hittinger C.T. (2013). *Saccharomyces* diversity and evolution: A budding model genus. Trends Genet..

[33] Liti G., Carter D.M., Moses A.M., Warringer J., Parts L., James S.A., Davey R.P., Roberts I.N., Burt A., Koufopanou V. (2009). Population genomics of domestic and wild yeasts. Nature.

[34] Sniegowski P.D., Dombrowski P.G., Fingerman E. (2002). *Saccharomyces cerevisiae* and *Saccharomyces paradoxus* coexist in a natural woodland site in North America and display different levels of reproductive isolation from European conspecifics. FEMS Yeast Res..

[35] Sampaio J.P., Gonçalves P. (2008). Natural populations of *Saccharomyces kudriavzevii* in Portugal are associated with oak bark and are sympatric with *S. cerevisiae* and *S. paradoxus*. Appl. Environ. Microbiol..

[36] Stefanini I., Dapporto L., Legras J.L., Calabretta A., Di Paola M., De Filippo C., Viola R., Capretti P., Polsinelli M., Turillazzi S. (2012). Role of social wasps in *Saccharomyces cerevisiae* ecology and evolution. Proc. Natl. Acad. Sci. USA.

[37] Stefanini I., Dapporto L., Berná L., Polsinelli M., Turillazzi S., Cavalieri D. (2016). Social wasps are a *Saccharomyces* mating nest. Proc. Natl. Acad. Sci. USA.

[38] Pieczynska M.D., Wloch-Salamon D., Korona R., de Visser J.A. (2016). Rapid multiple-level coevolution in experimental populations of yeast killer and nonkiller strains. Evolution.

[39] Bevan E.A., Makower M., Geerts S.J. (1963). The physiological basis of the killer character in yeast. Genetics Today; Proceedings of the XIth International Congress of Genetics, The Hague, The Netherlands, September 1963.

[40] Philliskirk G., Young T.W. (1975). The occurrence of killer character in yeasts of various genera. Antonie Van Leeuwenhoek.

[41] Diamond M.E., Dowhanick J.J., Nemeroff M.E., Pietras D.F., Tu C.L., Bruenn J.A. (1989). Overlapping genes in a yeast double-stranded RNA virus. J. Virol..

[42] Pfeiffer P., Radler F. (1984). Comparison of the killer toxin of several yeasts and the purification of a toxin of type K2. Arch. Microbiol..

[43] Ferrè F., Clote P. (2005). Disulfide connectivity prediction using secondary structure information and diresidue frequencies. Bioinformatics.

[44] Rodríguez-Cousiño N., Gómez P., Esteban R. (2017). Characterization of three new killer toxins from *Saccharomyces paradoxus* strains.

[45] Saitou N., Nei M. (1987). The neighbor-joining method: A new method for reconstructing phylogenetic trees. Mol. Biol. Evol..

[46] Felsenstein J. (1985). Confidence limits on phylogenies: An approach using the bootstrap. Evolution.

[47] Zuckerkandl E., Pauling L., Bryson V., Vogel H.J. (1965). Evolutionary divergence and convergence in proteins. Evolving Genes and Proteins.

[48] Tamura K., Stecher G., Peterson D., Filipski A., Kumar S. (2013). MEGA6: Molecular Evolutionary Genetics Analysis version 6.0. Mol. Biol. Evol..

[49] Esteve-Zarzoso B., Belloch C., Uruburu F., Querol A. (1999). Identification of yeasts by RFLP analysis of the 5.8S rRNA gene and the two ribosomal internal transcribed spacers. Int. J. Syst. Bacteriol..

[50] Špírek M., Poláková S., Jatzová K., Sulo P. (2015). Post-zygotic sterility and cytonuclear compatibility limits in *S. cerevisiae* xenomitochondrial cybrids. Front. Genet..

[51] Querol A., Barrio E., Ramón D. (1992). A comparative study of different methods of yeast strains characterization. Syst. Appl. Microbiol..

[52] Greig D., Borts R.H., Louis E.J., Travisano M. (2002). Epistasis and hybrid sterility in *Saccharomyces*. Proc. Biol. Sci..

[53] Liti G., Peruffo A., James S.A., Roberts I.N., Louis E.J. (2005). Inferences of evolutionary relationships from a population survey of LTR-retrotransposons and telomeric-associated sequences in the *Saccharomyces sensu stricto* complex. Yeast.

[54] Zhang H., Skelton A., Gardner R.C., Goddard M.R. (2010). *Saccharomyces paradoxus* and *Saccharomyces cerevisiae* reside on oak trees in New Zealand: Evidence for migration from Europe and interspecies hybrids. FEMS Yeast Res..

[55] Wickner R.B., Icho T., Fujimura T., Widner W.R. (1991). Expression of yeast L-A double-stranded RNA virus proteins produce derepressed replication: A ski- phenocopy. J. Virol..

[56] Rowley P.A., Ho B., Bushong S., Johnson A., Sawyer S.L. (2016). XRN1 Is a Species-Specific Virus Restriction Factor in Yeasts. PLoS Pathog..

[57] Fujimura T., Esteban R. (2011). Cap-snatching mechanism in yeast L-A double-stranded RNA virus. Proc. Natl. Acad. Sci. USA.

[58] McBride R.C., Boucher N., Park D.S., Turner P.E., Townsend J.P. (2013). Yeast response to LA virus indicates coadapted global gene expression during mycoviral infection. FEMS Yeast Res..

[59] Lukša J., Ravoitytė B., Konovalovas A., Aitmanaitė L., Butenko A., Yurchenko V., Serva S., Servienė E. (2017). Different Metabolic Pathways Are Involved in Response of *Saccharomyces cerevisiae* to L-A and M Viruses. Toxins.

[60] Conde J., Fink G.R. (1976). A mutant of *Saccharomyces cerevisiae* defective for nuclear fusion. Proc. Natl. Acad. Sci. USA.

[61] Chou J.Y., Hung Y.S., Lin K.H., Lee H.Y., Leu J.Y. (2010). Multiple molecular mechanisms cause reproductive isolation between three yeast species. PLoS Biol..

[62] Ramírez-Garrastacho M., Esteban R. (2011). Yeast RNA viruses as indicators of exosome activity: Human exosome hCsl4p participates in RNA degradation in *Saccharomyces cerevisiae*. Yeast.

[63] Fujimura T., Esteban R., Esteban L.M., Wickner R.B. (1990). Portable encapsidation signal of the L-A double-stranded RNA virus of *S. cerevisiae*. Cell.

[64] Sambrook J., Fritsch E.F., Maniatis T. (1989). Molecular Cloning: A Laboratory Manual.

[65] Zuker M., Mathews D.H., Turner D.H., Barciszewski J., Clark B.F.C. (1999). Algorithms and thermodynamics for RNA secondary structure prediction: A practical guide. RNA Biochemistry and Biotechnology.

