# Supplementary material for: Variation and Distribution of L-A Helper Totiviruses in Saccharomyces sensu stricto Yeasts Producing Different Killer Toxins
Source: Toxins (Basel). 2017 Oct 11;9(10):313. doi: 10.3390/toxins9100313 (PMC5666360; doi:10.3390/toxins9100313)
Supplement: Supplementary file 1 [file toxins-09-00313-s001.pdf]

Supplementary Materials: Variation and Distribution of L-A Helper Totiviruses in *Saccharomyces sensu stricto* Yeasts Producing Different Killer Toxins

Nieves Rodríguez-Cousiño, Pilar Gómez and Rosa Esteban

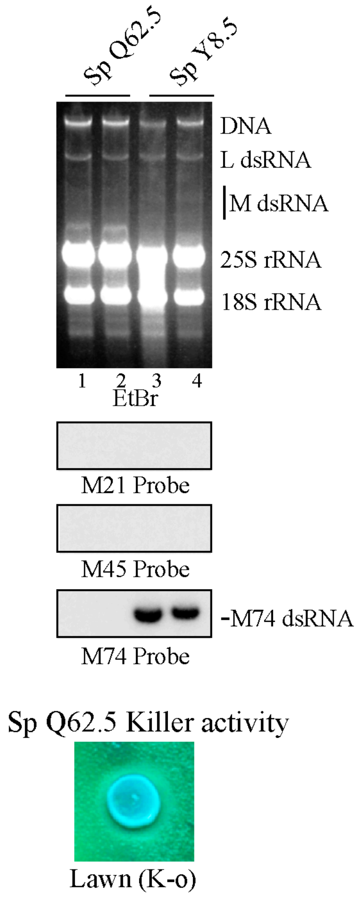

Figure S1. Analysis of M dsRNAs from Strains Sp Q62.5 and Sp Y8.5 by Northern hybridization. The upper panel shows an ethidium bromide-stained agarose gel with total nucleic acids prepared from two colonies of each strain. After transference to a nylon membrane three specific probes were used for hybridization that recognize M21 (from strain Sp T21.4), M45 (from strain Sp N-45) or M74 (from strain Sp Q74.4). The autoradiograms are shown. Note that none of the probes recognize dsRNA in strain Q62.5 (lanes 1 and 2), whereas the dsRNA in strain Sp Y8.5 (lanes 3 and 4) hybridize with the M74-specific probe. The lower panel shows killer activity of Strain Sp Q62.5 over a lawn of a K-o strain.

		1	2	3	4	5	6	7	8	9	10	11	12	13	14
1:	Sk IFO1082	100.0	73.0	74.3	73.6	73.4	73.7	73.5	73.1	73.0	73.7	73.7	73.5	73.6	72.8
2:	Sc L-A	73.0	100.0	74.1	73.5	75.5	74.7	73.5	74.0	74.1	73.9	74.1	73.7	74.1	73.9
3:	Sc L-A-2	74.3	74.1	100.0	77.9	74.9	75.9	75.5	75.8	75.6	75.6	75.8	75.8	75.5	75.4
4:	Sc L-A-lus	73.6	73.5	77.9	100.0	75.1	76.2	75.0	75.9	75.8	75.8	75.6	75.9	76.1	76.0
5:	Sp N-45	73.4	75.5	74.9	75.1	100.0	76.5	76.5	76.2	76.6	76.3	76.6	76.1	76.4	76.0
6:	Sk FM1183	73.7	74.7	75.9	76.2	76.5	100.0	79.3	78.9	79.4	79.1	79.1	79.6	79.2	78.9
7:	Sp DBVPG4650	73.5	73.5	75.5	75.0	76.5	79.3	100.0	89.5	90.2	90.1	90.4	90.3	82.7	81.4
8:	Su 10560	73.1	74.0	75.8	75.9	76.2	78.9	89.5	100.0	92.3	91.7	90.3	90.1	81.9	81.7
9:	Sp CECT1939	73.0	74.1	75.6	75.8	76.6	79.4	90.2	92.3	100.0	92.8	91.4	91.5	82.0	81.7
10:	Sp Q74.4	73.7	73.9	75.6	75.8	76.3	79.1	90.1	91.7	92.8	100.0	91.1	91.6	82.1	81.9
11:	Sp Q62.5	73.7	74.1	75.8	75.6	76.6	79.1	90.4	90.3	91.4	91.1	100.0	91.6	82.5	81.8
12:	Sp CECT11143	73.5	73.7	75.8	75.9	76.1	79.6	90.3	90.1	91.5	91.6	91.6	100.0	81.9	81.7
13:	Sp T21.4	73.6	74.1	75.5	76.1	76.4	79.2	82.7	81.9	82.0	82.1	82.5	81.9	100.0	89.8
14:	Sp L-A-28	72.8	73.9	75.4	76.0	76.0	78.9	81.4	81.75	81.7	81.9	81.8	81.7	89.8	100.0

Figure S2. Nucleotide sequence identity of L-A variants obtained by a ClustalW comparison. The sequences used are 4560 nt long. Strains belonging to the same species are colored with the same background: light blue (*S. kudriavzevii*), red (*S. paradoxus*), yellow (*S. cerevisiae*) and green (*S. uvarum*). Note that strain #6 (Sk FM1183) carries an L-A variant more closely related to *S. paradoxus* variants than to the one in strain Sk IFO1082 (#1) from a Far Eastern collection. The same is true in the case of Sp N-45 (#5) from Far East: its L-A variant is quite different from other variants of European origin in the same species. The two L-A variants more closely related are the ones present in Sp Q74.4 (#10) and Sp CECT1939 (#9) with 92.8% identity, though they carry different M viruses (boxed). The most divergent ones are those from Sk IFO1082 (#1) and Sp L-A-28 (14) with only 72.8% identity (boxed).

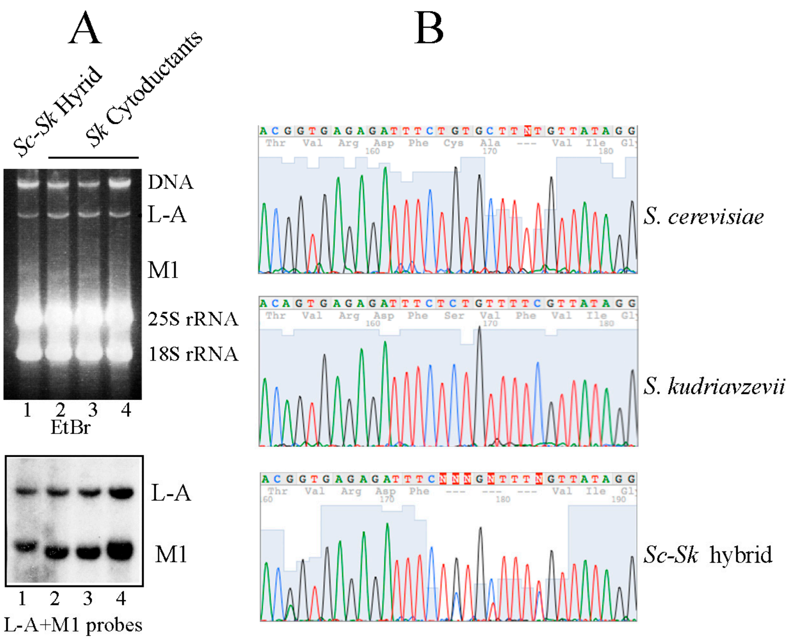

Figure S3. (A) Detection of L-A and M1 in K1 killer cells of a *S. cerevisiae*/*S. kudriavzevii* hybrid diploid or *S. kudriavzevii* cytoductants. RNAs from 1 hybrid diploid clone (lane 1) or 3 independent S. *kudrivzevii* cytoductants (lanes 2-4) obtained by horizontal cross-transmission of K1 viruses from *S. cerevisiae* were separated on an agarose gel (upper panel) and transferred into a nylon membrane for Northern hybridization with a mixture of M1- and L-A-specific probes (lower panel). **(B)** 5.8S-ITS rDNA sequencing of *S. cerevisiae, S. kudriavzevii* or a hybrid diploid of both species. The technique shows that the hybrid DNA (lower panel) contains a mixture of nucleotides (N) in few positions in the DNA fragment analyzed.

Table S1. Cross-killing activity of Saccharomyces killer strains. Cross-killing assays were done using 2 µl drops of saturated cultures of *S. paradoxus* strains T21.4 (K21), S28 (K28), N-45 (K45), Q62.5 (K62) and Q74.4 (K74) and *S. cerevisiae* strains 2403 (K1) and 1137 (K2) (vertical columns) that were spotted on MB plates seeded with lawns of the same strains as well as the sensitive strain 5X47 (horizontal columns). Clear killing halos are denoted by +, weak killers as $+^{w}$.

	**K21**	**K28**	**K45**	**K62**	**K74**	**K1**	**K2**
K21							
K28					$+^{w}$	+	+
K45		+			+	+	
K62					$+^{w}$	+	+
K74	$+^{w}$	+				+	+
K1	+	+	+	$+^{w}$			+
K2	+	$+^{w}$	+			+	
5X47	+	+	+	+	+	+	+
